# Role of autophagy in cancer-associated fibroblast activation, signaling and metabolic reprograming

**DOI:** 10.3389/fcell.2023.1274682

**Published:** 2024-01-03

**Authors:** Dyana Sari, Devrim Gozuacik, Yunus Akkoc

**Affiliations:** ^1^ Koç University Research Center for Translational Medicine (KUTTAM), Istanbul, Türkiye; ^2^ Department of Medical Biology, School of Medicine, Koç University, Istanbul, Türkiye; ^3^ Department of Biotechnology, SUNUM Nanotechnology Research and Application Center, Istanbul, Türkiye

**Keywords:** cancer-associated fibroblasts (CAFs), autophagy, tumor microenvironment (TME), fibroblast transdifferentiation, cancer

## Abstract

Tumors not only consist of cancerous cells, but they also harbor several normal-like cell types and non-cellular components. cancer-associated fibroblasts (CAFs) are one of these cellular components that are found predominantly in the tumor stroma. Autophagy is an intracellular degradation and quality control mechanism, and recent studies provided evidence that autophagy played a critical role in CAF formation, metabolic reprograming and tumor-stroma crosstalk. Therefore, shedding light on the autophagy and its role in CAF biology might help us better understand the roles of CAFs and the TME in cancer progression and may facilitate the exploitation of more efficient cancer diagnosis and treatment. Here, we provide an overview about the involvement of autophagy in CAF-related pathways, including transdifferentiation and activation of CAFs, and further discuss the implications of targeting tumor stroma as a treatment option.

## Introduction

Tumors were not only made of cancerous cells, but also consisted of several different types of non-cancerous cells and non-cellular components, forming the tumor microenvironment (TME). Historically, a vast majority of cancer studies focused on the phenotypic and molecular changes in cancer cells. However, studies in the last decade indicated that eradicating this deadly disease requires going beyond focusing only on cancer cells, and targeting other components such as TME might provide further advantages for diagnosis and treatment of cancer.

TME has a complex structure and was identified to contain different cell types and non-cellular material. Cellular compartments of the TME mainly consist of tissue-resident cell types, including fibroblasts, endothelial cells, or immune cells, including T and B lymphocytes and macrophages (Bussard et al., 2016). ECM, growth factors, cytokines, antibodies, and metabolites were associated with the non-cellular portion of the TME (Lu et al., 2012). Composition of the TME was strictly associated with cancer type, disease stage, and treatment. In addition, existence or recruitment of specific cell types to tumor sites might affect tumor phenotype, indicating the importance of intercellular crosstalk in the TME (Hinshaw and Shevde, 2019). The connection between stroma cells, cancer cells and stroma components was facilitated either by their physical contacts or through secreted factors. Cell-to-cell interactions through gap junctions or receptor-ligand interactions were crucial in this connection. Additionally, soluble factors, including cytokines, antibodies, and metabolites, contribute to the connection and communication between cancer cells and the TME.

Mechanical characteristics of the TME were also important. For instance, architecture of the ECM components, such as collagen, and its reorganization by several ECM remodeling enzymes, such as matrix metalloproteases (MMPs) were addressed in this context (Egeblad and Werb, 2002). These mechanical and physical features of the ECM surely affect the tumor phenotype. Indeed, ECM stiffness may facilitate the activation of certain TME cell types or create a concentration gradient for certain growth factors. Through these and other functions, TME components were found to be closely associated with tumor phenotype, aggressiveness, and treatment responses (Akkoc et al., 2021; Akkoc and Gozuacik, 2021).

Among the TME players, cancer-associated fibroblasts (CAFs) were one of the most abundant cellular components in most solid tumors (Kalluri, 2016). Fibroblasts generate most of the structural proteins of the ECM and were responsible for the secretion of several growth factors and cytokines. Different physiological and pathological stimuli may structurally and phenotypically change the phenotype of these fibroblasts, leading to their differentiation or activation (Öhlund et al., 2017). Several studies provided evidence that these specific types of active fibroblasts, CAFs, contributed to the initiation and promotion of tumor growth and affect tumor behavior under different contexts (Orimo et al., 2005; D’Arcangelo et al., 2020).

In this review, we explore recent advances about the role of autophagy in CAF phenotype and function and discuss how it contributes to tumor-stroma interactions. Studies on the involvement of autophagy in CAF differentiation and activation, CAF-mediated tumor progression, and cancer treatment will be analyzed. The potential therapeutic outcomes of these findings will be discussed.

## Mechanisms and regulation of autophagy

Autophagy is an evolutionarily conserved catabolic process whose activity is necessary for the maintenance of cellular homeostasis. Macroautophagy, microautophagy, and chaperone-mediated autophagy were recognized as the three major types of autophagy (Marzella et al., 1981; Mizushima et al., 2011; Kaushik and Cuervo, 2018). Macroautophagy (referred to herein as autophagy) was responsible for the recruitment and degradation of intracellular components in the lysosome either in a non-selective or selective manner. It allows the removal of unfolded proteins or misfolded protein aggregates, damaged or malfunctioning organelles, such as mitochondria or pathogens, such as intracellular bacteria (Okamoto et al., 2009; Roberts et al., 2013; Ohsumi, 2014). Autophagy is active under basal conditions. However, extrinsic or intrinsic stimuli or stress further upregulate autophagy. Various inducers, including growth factor deprivation, oxidative stress, hypoxia, ER stress, and metabolic stress, lead to the activation of autophagy through the core autophagy pathway. More than 40 core autophagy genes, known as autophagy-related genes (ATGs), were involved in the regulation of autophagy. Protein products of these genes form several protein complexes that were responsible for the control of autophagy signaling. The process could be divided into different stages: Upstream signaling, initiation and autophagosome formation, and fusion with lysosomes.

The mammalian target of rapamycin complexes (mTORC1 and 2) were the most significant upstream regulators of autophagy. The vital element of both mTOR protein complexes was the core serine/threonine kinase, or mTOR kinase. These protein complexes were important regulators of several cellular events such as cell growth, cell cycle, and protein synthesis. In addition to these, they also help to coordinate the activation of cellular catabolic process autophagy with the action of these vital cellular anabolic pathways. mTOR complexes were active and the autophagic machinery was inhibited when growth conditions were favorable. The downstream Atg1/Ulk1 autophagy-related kinase complex was regulated by mTORC1 (Mizushima et al., 2011). ATG13 and ULK1/2 were phosphorylated by mTOR in nutrient-rich environments, and the phosphorylation of FIP200 was negatively linked with their activity. Conversely, mTOR targets undergo dephosphorylation in response to nutrient starvation, and ATG13 binds to FIP200 and ULK1/2. Next, FIP200 and the FIP200-ULK1-ATG13 complex were phosphorylated by ULK1/2 (Laplante et al., 2012). Thus, the class-III phosphatidylinositol 3-kinase (PI3K) complex, which comprises the lipid kinase Vps34, was regulated by the active Atg1/ULK1 complex. Downstream of the ULK complex, VPS34 complex I, which includes VPS34, Beclin-1, ATG14, and VPS15, produces phosphatidylinositol-3-phosphates (PI3P) on membranes (Kroemer et al., 2010). Autophagic activity relies on cellular membrane sources and organelles, including the ER, Golgi, endosomes, and mitochondria, as contributors to forming membranes. Notably, *de novo* phospholipid synthesis was also required for autophagosome elongation (Tooze and Yoshimori, 2010). Phagophores were small initial lipid-based structures that elongate into large double-membrane autophagosomes following rapid elongation and closure. PI3P was a phosphoinositide that serves as a tag on membranes and allows recruitment of other factors involved in autophagosome formation (Yang et al., 2010). PI3P at the nascent autophagosome was recognized by ATG18/WIPI, WD-repeat containing proteins (Polson et al., 2010). They were other important players in autophagosome formation which regulate autophagic activity through the recruitment of two ubiquitin-like recruitment systems. In addition, a multi-pass transmembrane protein ATG9, was involved in the transport of membranes to forming autophagosomes by recruiting membranes from late endosomes and the trans-Golgi network (Noda et al., 2000).

Two ubiquitination-like conjugation systems, namely, ATG12-5-16L1 and ATG8 systems, were required during the autophagosome membrane elongation (Mizushima et al., 2011). In the first conjugation system, with the help of ATG7 (E1-like enzyme) and ATG10 (E2-like enzyme) ATG12 was conjugated to ATG5 proteins. Following addition of ATG16L protein to the complex facilitates the covalent conjugation of ATG12 to the lysine 130 residue (K130) of ATG5. Hence the formation of an autophagy-related 800-kDa protein complex was established following oligomerization of ATG16L proteins (Mizushima et al., 2011). The second system involves the conjugation of ATG8/LC3 to a lipid molecule, generally to a phosphatidylethanolamine (PE). Formation of ATG12-5-16L1 complexes from the first system possesses an E3-like enzyme activity that was required for the second ubiquitination-like conjugation system. The carboxyl terminus of LC3 protein was cleaved by Atg4 cysteine proteases. After cleavage, a glycine residue was exposed, and a free cytosolic form of the protein called LC3-I was generated. Then, membrane-bound form of autophagic LC3-II, where LC3-I was conjugated to a PE, was obtained with the help of ATG7 and ATG3 E2-like enzymes. (Hanada et al., 2007; Nakatogawa et al., 2007). LC3-II form was associated with mature autophagosomes, and it was commonly used as a marker of autophagy. Thus, distribution and number of autophagosomes could be identified by using LC3-II as a marker in autophagic activity analyses.

Initially, autophagy was described as a nonselective degradation pathway yet, recent studies showed that different autophagy receptors were capable of recognizing specific cargo molecules, underlining the fact that autophagy might be selective (Kraft et al., 2010; Ohsumi et al., 2014). Cargo recognition molecules or autophagy receptors such as SQSTM1/p62, NDP52, and NBR1 in mammals share specific cargo-interacting (ubiquitin-dependent, e.g., UBA or ubiquitin-independent, e.g., PB1) domains and LC3-interacting motifs (LIR) (Lamark et al., 2009; Kraft et al., 2010). These receptors form bridges between selected cargo molecules and ATG8 proteins, leading to their selective degradation. Thus, their cellular levels were generally downregulated following autophagy activation due to their delivery to autolysosomes together with the cargo. Hence, degradation of autophagy receptors was also another commonly used marker of autophagic activity. After completion and closure of autophagic vesicles, the last stage involves their fusion with late endosomes or lysosomes. ESCRT complexes, ATG proteins, SNAREs, Rab GTPases, Rab-related proteins and dynein-mediated transport of autophagosomes along the microtubules were required for the fusion of these two distinct compartments (Jäger et al., 2004; Zhang et al., 2018). Finally, the cargo inside the autophagosome was delivered to the lysosomal lumen and degraded by the action of hydrolytic enzymes in this compartment to maintain cellular homeostasis.

## Cancer-associated fibroblasts (CAFs) in the TME

### Activation and differentiation of CAFs in TME

CAFs differentiate from normal tissue cells in the TME, including fibroblasts. When biopsy specimens were examined, CAFs defined as cells that were negative for epithelial, endothelial, and leukocyte markers, and with a distinct fibroblast-like elongated morphology. Genetic changes observed in cancer cells were not shared by CAFs. Yet, epigenetic alterations seem to be responsible for inducing phenotypic and functional changes in these cells (Sahai et al., 2020).

Cancer-associated fibroblasts (CAFs) were activated fibroblasts that were associated with cancer cells and their transformation was facilitated by a variety of TME components including growth factors, cytokines, and chemokines (Kalluri et al., 2006). Different characteristics, such as the cells from which they originate, tissue position, shape, and the lack of lineage markers for other cells, led to differentiate CAFs (Kalluri et al., 2016). Activated CAFs have higher metabolic levels, increased proliferation and migration capacities in comparison to tissue-resident naive counterparts (Kobayashi et al., 2019). These variations enable CAFs to adjust to the TME, hence reinforcing their respective functions in the progression of cancer.

CAF populations might emerge from a single-cell source or derive from a number of cell types (De Wever et al., 2004). Tissue-resident naive fibroblasts, adipocytes, and mesenchymal stem cells were identified as major reservoirs of CAFs in the TME (Pavlides et al., 2012). The source of CAF differentiation appears to be tissue- and context-dependent (Bergers and Song, 2005; Calvo et al., 2013; Gascard and Tlsty, 2016). For example, mesenchymal stem cells (MSCs) can differentiate into CAFs. Bone marrow-derived MSCs that were recruited to the TME for tissue repair may serve as progenitor cells for CAFs (Quante et al., 2011; Weber et al., 2015). Stellate cells which were unique tissue-resident fibroblasts, under specific circumstances, could be stimulated and converted into CAFs. For instance, different studies showed that in response to TGF-β, IL-1, PDGF, and other stimuli, quiescent pancreatic stellate cells (PSCs) and hepatic stellate cells (HSCs) could develop a CAF phenotype and carry out their respective roles (Yin et al., 2013; Tan et al., 2020). Epithelial and endothelial cells could be transdifferentiated CAFs via EMT (Zeisberg et al., 2007; Fisher et al., 2015) and adipocytes could be transformed into CAFs (Tang et al., 2020).

In line with the view describing cancer as a non-healing, chronic wound, similarities between wound healing and tumor-stroma interactions were observed (Wang et al., 2017b). For instance, myofibroblasts were a differentiated form of naïve fibroblasts that gain a contractile phenotype following increased expression of α-smooth muscle actin (α-SMA) during wound healing process. During wound healing, myofibroblasts were transient (Grotendorst et al., 2004). Otherwise, persistent inflammation and continued cell division could lead to failed tissue repair and excessive fibrosis (Wynn, 2008). In contrast to the wound healing process, CAFs remained constantly active within the tumor contributing to chronic inflammation and in some cancer types promote further tissue fibrosis. Several studies showed that activated fibroblasts and CAFs initiated and promoted tumor growth (Orimo et al., 2005).

**FIGURE 1:**
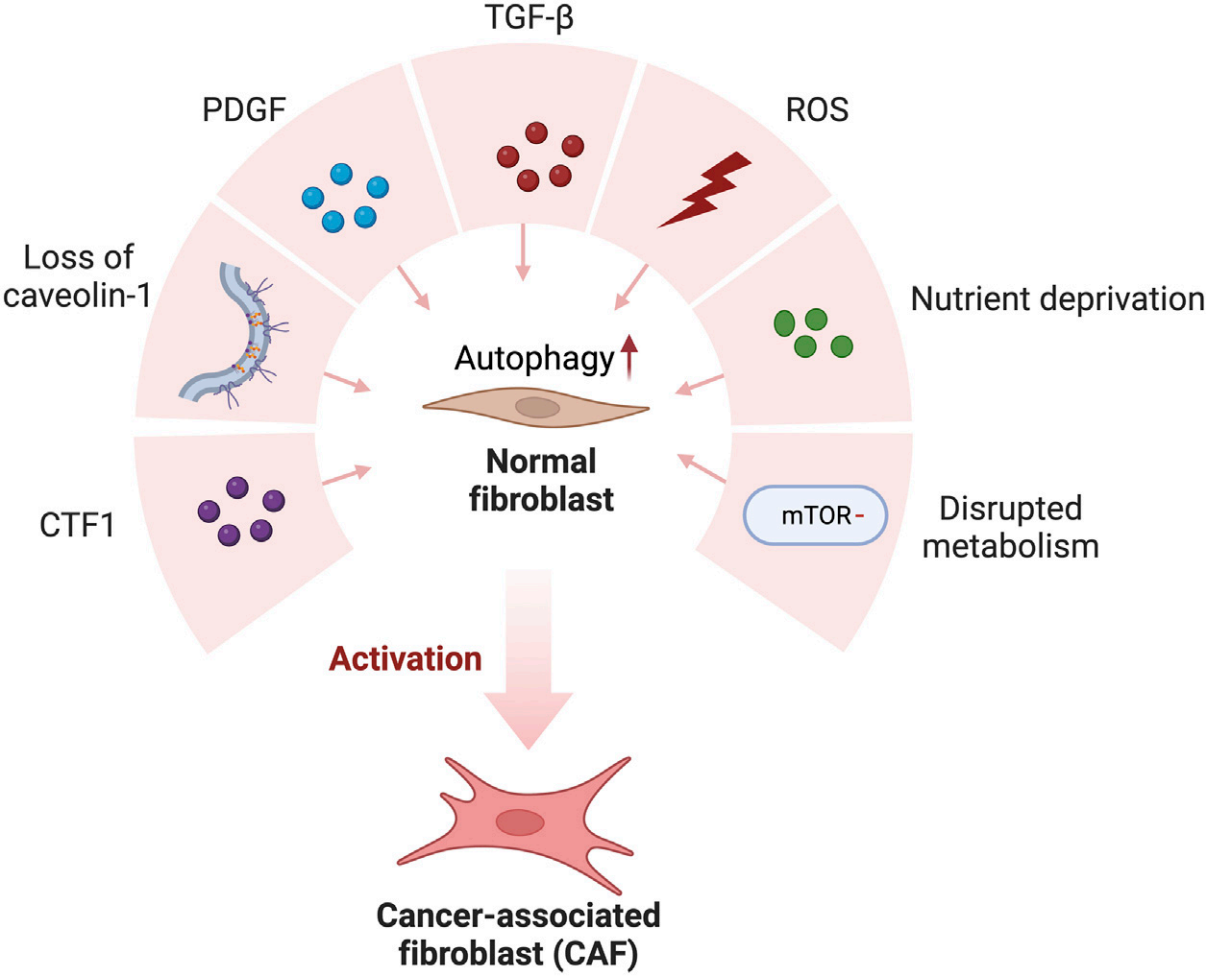
Cancer-derived secreted factors and the TME favor normal fibroblast conversion to cancer-associated fibroblasts (CAFs). Cancer-derived secretion of CTF1, PDGF, TGF-β and ROS and Loss of caveolin-1, nutrient deprivation, and disrupted metabolism favor autophagic activation in normal fibroblasts. As a result of high autophagic activity, normal fibroblasts transform into CAF in the tumor microenvironment.

The interaction between TME components, e.g., cancer cells and other cellular and non-cellular components, was crucial for the activation and differentiation of CAFs (Figure 1). Indeed, CAF activation and differentiation generally rely on physical factors and paracrine factors. For instance, in a pancreatic cancer model, CAF activation and its interaction with cancer cells were facilitated in both contact-dependent and paracrine manner. CAFs in cancer-prone areas were referred to as myofibroblastic CAFs (myCAFs), and they show high TGF-β-mediated α-SMA expression and have a contractile phenotype. However, an inflammatory phenotype, with a high expression of IL6 and a low expression of αSMA (ACTA2) has been associated with the inflammatory CAFs (iCAFs). myCAFs were shown to interact with cancer cells via juxtacrine/contact-dependent interactions, whereas iCAFs tend to interact with cancer cells via inflammatory cytokines (Öhlund et al., 2017; Elyada et al., 2019). Besides these two major CAF subgroups, a subset of CAF expressing CD74 and major histocompatibility complex class II (MHC-II) were also discovered and named “antigen-presenting CAFs” (apCAFs). Furthermore, apCAFs were found to directly ligate and stimulate naive CD4^+^ T cell differentiation into regulatory T cells (Tregs) in an antigen-specific manner (Elyada et al., 2019; Huang et al., 2022). The discovery of apCAFs raises the possibility that particular subgroups may act as a mediator for the immunomodulatory effect of CAFs.

These distinct CAFs types operate through different signaling pathways in tumors. Secretion profiles seem to determine which CAF type will be formed in tumor areas. For example, secretion patterns of CAFs residing at the periphery of tumors diverged: Secretion of IL6 and some other inflammatory mediators, including IL11 and LIF, became more prominent in these tumor areas (Öhlund et al., 2017). Tumor-secreted TGF-β and IL1 promoted the formation of two distinct CAF subtypes with myofibroblastic and inflammatory phenotypes in pancreatic cancer 3D cell culture models. IL1/LIF/JAK/STAT signaling has been shown to support the formation of inflammatory CAF, while TGF-β has the opposite effect by decreasing the expression of IL1R and stimulating the formation of myofibroblast subtypes (Biffi et al., 2019).

Cell-to-cell and juxtacrine interactions have also been observed in the TME of patients with cancer. In a study, physical interactions between breast cancer cells and fibroblasts triggered CAF formation via Notch signaling (Strell et al., 2019). A similar activation pattern has been reported in squamous cell cancers when Notch signaling was activated (Procopio et al., 2015). These studies suggest that the Notch signaling pathway was important for tumor-stroma interactions and the conversion of fibroblasts into CAFs. In another example of a paracrine interaction, IL1 has been shown to control CAF formation through the activation of STAT transcription factors, NF-κB activation and subsequent IL6 secretion (Sanz-Moreno et al., 2011). Here, suppression of IL1 signaling, NF-κB signaling and increased IL6 expression have been observed (Biffi et al., 2019). Furthermore, JAK/STAT signals were reported to regulate CAF formation by regulating the cytoskeleton and histone acetylation (Albrengues et al., 2015).

As mentioned above, several inflammatory cytokines and chemokines play important roles in the activation of CAFs. TGF-β family ligands and lipid mediators were by far one of the most studied signaling molecules in the context of cancer microenvironment (De Wever et al., 2004; Calvo et al., 2013; Foster et al., 2017). These mediators convey signals to SMAD transcription factors and serum response factors (SRFs) promoting fibroblast activation and expression of CAF markers such as α-SMA resulting in contractility (Tomasek et al., 2002). A member of the TGF-β superfamily member, Nodal, facilitated the differentiation of tissue-resident fibroblasts into CAFs by activating Snail and TGF-β signaling (Z. Li et al., 2019). TGF-β was shown to induce autophagy in fibrotic diseases via SMAD3 signaling. Downregulation of MYST1, a histone acetyltransferase, regulated the expression of autophagic genes, including ATG7 and Beclin-1, further enhancing the release of collagen (Zehender et al., 2021).

Besides paracrine signaling pathways, physical changes in the ECM were reported to affect CAF formation (Calvo et al., 2013; 2015). For example, hyper-proliferation of transformed epithelial cells resulted in the activation of MRTF-SRF/YAP1-TEAD-mediated transcription, leading to fibroblast stretching *in vitro* (Calvo et al., 2013; Ao et al., 2015; Foster et al., 2017). These transcription factors control the expression of many genes associated with the CAF phenotype, including cysteine-rich angiogenic stimulator 61 (CYR61, CCN1) and connective tissue growth factors (CTGF, CCN2) (Foster et al., 2017). The contractile phenotype was congruent with the cytoskeleton, and the presence of these factors increased tissue stiffness and stimulated SRF- and YAP1-dependent transcriptional programs. Under these conditions, CAFs entered a self-sustaining positive feedback loop and remained active (Calvo et al., 2013).

Physiological and genetic stress factors were reported to stimulate fibroblast activation as well. For instance, heat shock factor-1 (HSF1), which responds to protein misfolding, was required for CAF formation (Scherz-Shouval et al., 2014; Ferrari et al., 2019). Dickkopf-3 (DKK3) was an HSF1 effector that interacts with common signaling pathways to sustain the protumorigenic activity of CAFs. Yes-associated protein (YAP)/transcriptional coactivator with PDZ-binding motif (TAZ) degradation was decreased as a result of HSF1-dependent DKK3 upregulation, which in turn promoted ECM remodeling of cancer cells (Ferrari et al., 2019). Moreover, double-strand DNA damage stimulated CAF formation by promoting secretion of IL6 and TGF-β family ligand activin-A (Gascard and Tlsty, 2016).

### CAF markers

Markers related to the CAF phenotype have been studied extensively (Table 1). Since CAFs have a high degree of plasticity, CAF cell population might be highly heterogeneous (Nurmik et al., 2020). Various biomarkers have been used to distinguish and isolate CAFs from fibroblasts and mesenchymal stem cells. For instance, fibroblast activation protein-α (FAP-α), α-SMA, platelet-derived growth factor receptor (PDGFRα/β) and vimentin were highly expressed in CAFs (Guo et al., 2008). Other commonly observed markers included myosin light chain-9 (MYL9), myosin light chain kinase (MYLK), matrix metalloproteinase 2 (MMP2), decorin (DCN) and collagen type I-α2 (COL1A2) were also determined (Nishishita et al., 2018; Nurmik et al., 2020). Therefore, detection of a collection of markers should be combined with morphological properties in order to correctly diagnose the CAF cells, and not to confuse them with stem cells and epithelial cells gaining mesenchymal phenotypes through de-differentiation (Manoukian et al., 2021), or cancer cells in EMT (Thiery et al., 2009).

**TABLE 1:** Positive and negative markers were used in the identification of CAFs.

**Highly expressed positive markers**	FAP-α	Garin-Chesa et al. (1990)
	α-SMA	Guo et al. (2008)
	PDGFRα/ß	Tejada et al. (2006)
	Vimentin	Zhou et al. (2014)
**Positive markers**	MYL9	Elyada et al. (2019)
	MYLK	Nishishita et al. (2018)
	MMP2	
	COL1A2	Öhlund et al. (2017)
**Other potential markers**	Transgelin	Planche et al. (2011), Elyada et al. (2019)
	Periostin	
	Podoplanin	Atsumi et al. (2008), Kerrigan et al. (2012)
	FSP1	Zeisberg et al. (2007)
**Negative markers**	EPCAM	Berdiel-Acer et al. (2014), Costa et al. (2018)

α-SMA, often referred to as smooth muscle aortic α-actin (ACTA2), was a protein that belongs to the actin family, a highly conserved group of molecules that were crucial for cell motility, structure, and integrity. α-SMA was recognized for its involvement in wound healing, where it regulates microfilament bundles and stress fibers, which were the crucial components for myofibroblast contractility (Nishishita et al., 2018). Production of new connective tissue in the wound area during the healing process, (known as the granulation tissue), was significantly influenced by mechanical forces depending on cellular contraction and α-SMA. CAFs upregulate the expression of this special protein, making it a general marker of myofibroblast and CAF populations. However, it has been hypothesized that α-SMA was differentially expressed across the various CAF subtypes (Öhlund et al., 2017).

FAP-α was another marker associated with CAFs. Almost 90% of epithelial carcinomas have elevated levels of FAP-α, one of the most highly expressed genes in the tumor stroma (Öhlund et al., 2014). It was a type II integral membrane protein that belongs to the serine protease family. Due to its dipeptidyl peptidase and collagenase activities, FAP-α has long been linked to tissue healing, fibrosis, and extracellular matrix breakdown by fibroblasts (Nurmik et al., 2020). Several studies have identified FAP-α as a marker of CAFs because of its high expression in the tumor stroma (Öhlund et al., 2014). Hence, FAP-α was now frequently used to identify prospective CAF populations, usually along with negative epithelial markers such as epithelial cell adhesion molecules EPCAMs (Berdiel-Acer et al., 2014). In contrast, in a study by Li et al., single-cell sequencing was used to characterize the transcriptome of the TME and showed that specific subpopulations of CAFs within the tumor microenvironment expressed variable levels of FAP-α and even absent in some tumor fibroblast subpopulations (H. Li et al., 2017).

Platelet-derived growth factor receptor (PDGFR) was a tyrosine kinase receptor found on the surface of fibroblasts, astrocytes, neural progenitors, and pericytes (Funa and Sasahara, 2014). PDGFRs were divided into two main groups, the first one was PDGFRα and the second one was PDGFRβ. Both were generally used as fibroblast markers. PDGFR overexpression has been observed in various tumor types, including gliomas, prostate cancer, and ovarian cancer (Heldin, 2013). The expression of platelet-derived growth factor (PDGF), a PDGFR ligand, was strongly associated with tumor growth and activated CAFs. For instance, PDGFB expression has been closely linked to the development of tumor stroma in melanoma (Forsberg et al., 1993), and PDGFA facilitated recruitment of PDGFR-positive stromal fibroblasts to tumor periphery in xenograft mouse models of lung carcinoma (Tejada et al., 2006). Different from the other markers, PDGFR was selectively and ubiquitously expressed on CAFs. Moreover, the expression of PDGFR and PDGF was less susceptible to environmental variables in the TME, such as hypoxia. Neither PDGF nor PDGFR exhibits considerable upregulation in CAF populations under hypoxic conditions (Madsen et al., 2015; Gascard and Tlsty, 2016). Therefore, PDGF and PDGFR might be used as reliable markers of CAFs.

An essential component in the development of the cytoskeleton network, particularly in cells of mesenchymal origin, was the type III intermediate filament protein, vimentin. This network was essential for positioning organelles within cells, cellular motility, and adhesion. Vimentin was considered as another CAF-associated marker (Nurmik et al., 2020). It was abundantly expressed in all types of fibroblasts (Hsia et al., 2016). Notably, in addition to fibroblasts, vimentin was also expressed in other cell types, including macrophages and adipocytes (Gascard and Tlsty, 2016). In addition, vimentin was produced by epithelial cells undergoing EMT, a process during which tumor cells exhibit increased expression of various mesenchymal markers. Therefore, although vimentin was considered a CAF-associated marker, its overall specificity as a marker, even for fibroblasts, was relatively low (Bergers and Song, 2005).

In addition to above-mentioned markers, other proteins were introduced as markers because of their higher expression levels in CAFs. For instance, fibroblast-specific protein 1 (FSP1), also known as S100 calcium-binding protein A4 (S100A4), was used in several studies for validation of the CAF phenotype of the investigated cells (Herrera et al., 2013; Lotti et al., 2013). However, recent studies placed some doubt on the specificity of FSP1, since it was found to be less accurate than FAP-α in identifying fibroblasts from primary tumor samples (Kahounová et al., 2018). In addition, FSP1 expression in fibroblasts was highly variable among different CAF subpopulations (Zeisberg et al., 2007).

Other potential markers such as transgelin (TGRLN) and periostin (POSTN) were also introduced in this context. These proteins were strongly expressed in fibroblasts and CAF cells (Planche et al., 2011). Nevertheless, like their more widely tested counterparts, their utility as CAF markers was hampered by limited selectivity, CAF subtype variations, and intracellular localization (H. Li et al., 2017). Another membrane-bound marker that was overexpressed in CAF populations was podoplanin (PDPN). Recent studies have suggested that this marker could potentially be used to identify pro-tumorigenic fibroblast subpopulations, as presence of PDPN-positive fibroblasts correlated with worse outcomes across multiple tumor types. Yet specificity problems exist, since high expression levels were noted in some epithelial tumor cells and inflammatory macrophages (Atsumi et al., 2008; Kerrigan et al., 2012). Of note, in addition to the notes above, the CAF phenotype could be diversified even under the same experimental conditions. Pancreatic stellate co-cultured with PDACs could differentiate both POSTN-positive CAFs (least protumoral) to myosin heavy chain 11 (MYH11) and PDPN-expressed (tumor-supportive) CAFs (De Wever et al., 2004).

Various negative markers were used to aid in the detection of fibroblast/CAF populations. Because of the lack of an exact definitive marker for CAFs and a lack of specificity for many of the positive markers used for CAF identification, negative selection was essential to rule out a variety of cell types that were frequently present in tumor tissue samples. Epithelial cell adhesion molecule (EPCAM) and smoothelin (SMTN) were markers often used to distinguish between epithelial and smooth muscle cells, respectively (Berdiel-Acer et al., 2014; Costa et al., 2018). Leukocytes and endothelial cells were examples of non-fibroblast cell types that were excluded using markers, including CD45, CD34, and CD11 (Takahashi et al., 2017).

Using genome-wide single-cell RNA sequencing (scRNAseq), studies suggested the existence of distinct CAF populations and led to a better understanding of CAF heterogeneity. For the first time in the literature, high ECM remodeling genes expressed extracellular matrix CAFs (eCAFs) were identified in gastric cancer (X. Li et al., 2022). apCAFs, which express MHCII and CD74 were identified with a similar approach (Elyada et al., 2019). In another study, they proposed that apCAFs could be originated from mesothelial cells by analyzing different stages of KIC tumors of pancreas again using scRNAseq by evaluating mesothelial lineage gene expression (Dominguez et al., 2020). CAFs were previously divided into 4 subtypes from CAF-S1 to CAF-S4 because of their distinct biomarker properties in breast cancer (Costa et al., 2018). However, further analyses by scRNAseq revealed that CAF-S1 can be further divided into 7 subtypes (from cluster 0 to cluster 7) (Kieffer et al., 2020). Other CAF subpopulations pan-myCAF, pan-dCAF, pan-iCAF, pan-pCAF, and pan-iCAF-2 were identified in melanoma, head and neck squamous cell carcinoma, and lung cancer by using similar analysis (Galbo et al., 2021).

CAFs could exert high plasticity due to the heterogeneity of cell origin, phase of activated fibroblastic state, activation signals and RNA profiles. Although the existence of universal markers to identify CAFs that were generated by different TME-derived factors remains elusive, all these observations allowed us a better understanding of diverse CAF phenotypes.

## Role of autophagy in CAF activation and signaling in the TME

One of the main factors influencing the development and spread of cancer was the dysregulation of particular signaling pathways in cancer cells. Dysregulation of signaling pathways could be modulated in many ways by the function of CAFs by creating a feedback loop between the tumor and the stroma that controls the initiation and progression of cancer. Autophagy was one of the hub pathways that seems critical in this crosstalk. In the next sections, we will summarize the role of autophagy in this context.

**FIGURE 2:**
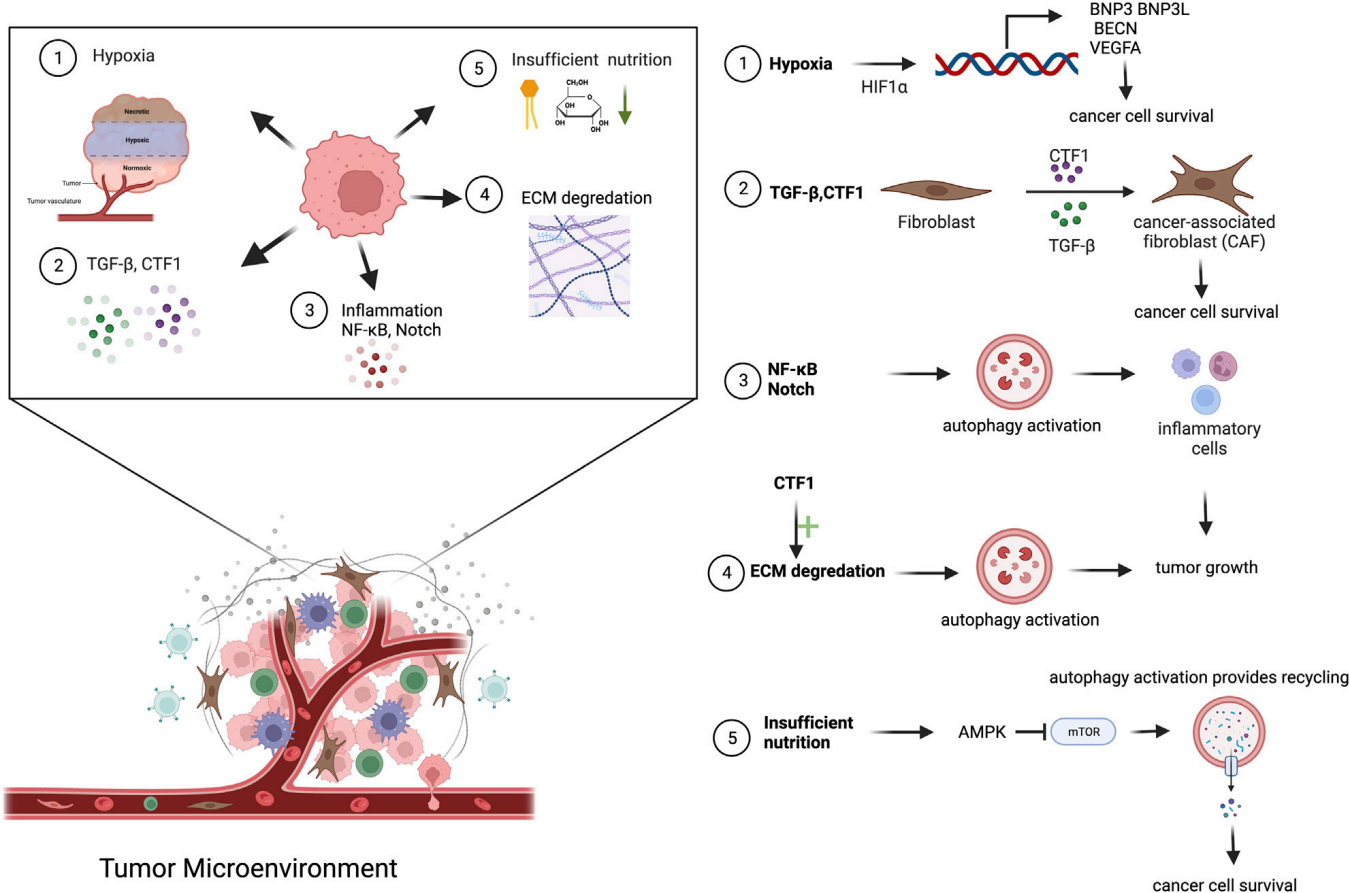
Cancer-derived secreted factors and autophagy activation in TME. Cancer cells secrete a diverse range of molecules into the TME. Mainly hypoxic condition, insufficient nutrition, NF-κB, TGF-β and CTF1 secretion, ECM degradation cause autophagy activation in TME.

The persistent autophagic activity was associated with differentiation of fibroblasts into CAFs. Conversely, inhibition of autophagy reduced the expression of myofibroblastic genes and restricted CAF formation (Bernard et al., 2020; Akkoc et al., 2023). Therefore, autophagic activity in the TME appears to be a key mechanism for fibroblast activation and CAF formation (Figure 2) (Akkoc and Gozuacik, 2021; Akkoc et al., 2023).

In tissues, myofibroblast differentiation and fibrosis were associated with cellular senescence, a cellular response to persistent stress. ROS were well-known inducers of senescence. A study was conducted using ROS scavengers, and the role of autophagy in both fibrosis and CAF differentiation was analyzed (Martinez-Outschoorn et al., 2011). Exposure of fibroblasts to ROS scavengers reduced ROS generation during serum starvation (an inducer of autophagy), blocked autophagy, and dramatically lowered the expression of senescence and myofibroblast differentiation markers. In another study, cancer-derived ROS switched on autophagy in the stroma, especially in CAFs, as a major stress-response mechanism (Narita et al., 2011). These activated CAFs have been found to supply high-energy substances, including ketone bodies and lactate, to cancer cells helping to maintain the energy needed for their proliferation (Martinez-Outschoorn et al., 2011; Narita et al., 2011).

Hypoxia was another significant stress condition faced by cells in the TME. Hypoxia-associated signaling was controlled by HIF1α transcription factors. Crosstalk between HIF1α and autophagy has been studied in different contexts. Hypoxia-induced HIF1α regulated the expression of BNIP3 and BNIP3L, which share atypical BH3 domains. Therefore, they could bind and occupy anti-apoptotic Bcl-2 and Bcl-xl, both of which were Beclin-1 interactors, eventually resulting in the accumulation of free Beclin-1 and consequent autophagy induction (Bellot et al., 2009; Mazure and Pouysségur, 2010). In a parallel study, under anaerobic conditions, binding of HIF1α, HIF2α and Nanog at the BNIP3L promoter was found to serve as an autophagic stimulus (Hasmim et al., 2017). Moreover, HIF1α was activated in breast cancer cells, leading to the activation of NF-κB signaling and concomitant activation of mitophagy in CAFs (Martinez-Outschoorn et al., 2010). Loss of BRCA1, a mutation of BRCA1, manifested in 45% of hereditary breast cancers was associated with low autophagic activity, leading to an increase in the level of HIF1α and leading to the production of ketone bodies and modulation of the autophagic activity (Salem et al., 2012). Elevated ATG5 and BNIP3 expression were found in stromal cells in an acidic microenvironment, which provided evidence that CAFs may use autophagy as a survival pathway under stress conditions (Wojtkowiak et al., 2012). Upon hypoxia, TGF-1 signaling was upregulated, contributing to conversion of tissue fibroblasts to CAFs. In this context, hypoxia increased the expression of MCT4 in parallel with TGF-1, which stimulated aerobic glycolysis in CAFs (Jena et al., 2022). All these studies demonstrate the importance of hypoxia as a stress factor in the TME and the role of autophagic activity under these conditions.

Prolonged starvation was accompanied by the activation of MTORC2, but not MTORC1, and was suggested to sustain autophagic activity upon starvation (Bernard et al., 2014). In this model, autophagic activity upon starvation correlated with elevated levels of fibroblast activation markers, including α-SMA and Col1a1. Loss of autophagic capacity, either through pharmacological or genetic inhibition, suppressed CAF differentiation. This phenomenon was associated with elevated secretion of connective tissue growth factor (CTGF) from the autophagic CAFs (Bernard et al., 2014). In another study, elevated CSL levels in CAFs resulted in the attenuation of autophagy through autophagy receptor p62 regulation (Goruppi et al., 2017; 2018). In contrast, increased autophagic activity caused a reduction in both p62 and RBPJ levels, which enabled CAF activation. In another example, lncRNAs have been found to regulate autophagy and CAF activation. LncRNA-CAF derived from oral squamous cell carcinoma cells via exosomes led to the elevation of IL33. IL33 levels were maintained by the lncRNA-CAF-mediated blockage of p62-associated lysosomal degradation of the factor. The expression levels of CAF activation-related molecules, including α-SMA and vimentin, were controlled by IL33 (Ding et al., 2018). In the same context, another lncRNA, MALAT1, has been implicated in the activation of the Akt/mTOR pathway, which eventually resulted in the activation of autophagy in gastric cancer cells. Inhibition of autophagy resulted in elevated secretion of IL6 and further led to the activation of CAFs (Z. Wang et al., 2021). Similar to IL6, TGF-β was also found to be responsible for both regulating autophagic activity and activation of CAFs. Levels of the CAF activation-related factors ATF6 and Nrf2 were found to be strictly controlled by autophagy, which was correlated with elevation of p62 levels (Kang et al., 2021). Reduction of proline biosynthesis and collagen production as a result of autophagy downregulation was associated with the lack of CAF activation (Bai et al., 2023). In another example, autophagic activity was required for CAF activation and it was maintained by an autocrine secretion loop involving the HMGB1 pathway. In this study, autophagy was more active in CAFs than in normal lung fibroblasts. Inhibition of CAF autophagy decreased the EMT and metastatic capacity of lung cancer cells through NF-κB signaling (Ren et al., 2021). In parallel, delivery of LMP1 via extracellular vehicles resulted in differentiation of normal fibroblasts into CAFs by activating the NF-κB signaling (Wu et al., 2020).

We recently identified a novel inducer of the CAF phenotype, namely, cardiotrophin-1 (CTF1) (Akkoc et al., 2023). Similar to TGF-β, cancer cell-derived CTF1 alone induced CAF phenotype demonstrated by α-SMA and vimentin upregulation, actin stress fiber formation and enhanced contractile properties in fibroblasts. In contrast, fibroblasts lacking functional autophagic machinery were not activated and they did not show the CAF phenotype. Off note, CTF1-mediated CAF induction mechanisms were different from that of TGF-β, since it was independent of IL6 involvement in our system. CTF1-related autophagy and CAF activation supported cancer cell migration and invasion *in vitro* and correlated with lymph node metastasis in patient-derived tissue samples. Hence, CTF1 was a novel component of cancer-TME crosstalk and an inducer of autophagy-dependent CAF formation and cancer metastasis (Akkoc et al., 2023).

As a catabolic pathway and stress response mechanism autophagy was also linked to metabolic reprogramming of the CAFs. Thus, we will summarize possible roles of autophagy in CAF metabolic shift.

## Role of autophagy in metabolic reprogramming of CAFs in TME

Changes in the balance between anabolic and catabolic processes, as well as reprogramming of the metabolic pathways were observed in cancer cells as well as in the components of the TME. Metabolic reprogramming of CAFs could be triggered and strengthened by direct intercellular interactions or paracrine signaling between the CAFs and cancer cells (Fiaschi et al., 2012). Metabolic reprogramming in CAFs, may become self-sustaining, potentially in part due to epigenetic remodeling of these cells (Becker et al., 2020).

Metabolic responses of CAFs might be related to intratumoral hypoxia and enhance their ability to survive. In CAFs, aerobic glycolysis seems to be a key component of cellular metabolism (Petherick et al., 2015). In this context, enhanced autophagy and catabolic activity were correlated with increased aerobic glycolysis, a phenomenon known as the Warburg effect in CAFs (Chaudhri et al., 2013; Zhang et al., 2018). The glycolytic pathway may become dominant during hypoxia through upregulation of the expression of glucose transporters and relevant enzymes (Pagano et al., 2014). Indeed, activated HIF1α signaling in fibroblasts was found to determine the volume and mass of the tumor without a significant increase in tumor angiogenesis. Similar outcomes were observed for NF-κB activation, another autophagy inducer (Chiavarina et al., 2010). Autophagy-assisted metabolic modifications in CAFs were suggested to play a crucial role in supporting cancer cell metabolism through the intercellular supply of critical energy substrates. Ketone bodies, fatty acids, and glutamine were counted as potential fuel sources that were provided by CAFs for mitochondrial respiration in anabolic cancer cells (Martinez-Outschoorn et al., 2011; Chaudhri et al., 2013; Zhang et al., 2018).

CAFs underwent metabolic reprogramming and support glycolysis and produce critical metabolites that fuel the cancer cells. For instance, TGF-β and PDGF have been found to favor aerobic glycolysis over oxidative phosphorylation in CAFs. This conversion was characterized by the downregulation of isocitrate dehydrogenase 3 (IDH3), which catalyzes the first oxidative reaction in the TCA cycle. It was shown that miR-424 was responsible for the downregulation of IDH3A. Yet, gene methylation might also contribute to IDH3A downregulation in CAFs. Downregulation of IDH3 reduces the effective amount of α-ketoglutarate (α-KG), by lowering the ratio of α-KG to fumarate and succinate, which then inhibits PHD2 and stabilizes the HIF1α protein. Indeed, loss of IDH3 expression was associated with CAFs in tumor tissues, whereas overexpression of IDH3 inhibits the conversion of fibroblasts to CAFs. Hence, expression level of IDH3 might contribute to the fibroblast-CAFs switch (Zhang et al., 2018).

Cancer cells mostly rely on aerobic glycolysis. However, CAF-derived energy sources such as l-lactate, glutamine, free fatty acids, and ketone bodies can fuel mitochondrial biogenesis. Concomitantly, to produce the required energy, oxidative metabolism was activated (Chaudhri et al., 2013; Zhang et al., 2018). Under these circumstances, aerobic glycolysis capacity and autophagy of cancer cells were sustained by CAF-derived metabolites. Tumor cells deaminate glutamine in the mitochondria and produce ammonia as a by-product. Tumor-derived ammonia was shown to stimulate autophagic activity in cancer cells and surrounding CAFs (Li et al., 2021). In another study, the glycolytic capacity of lung cancer tumors correlated with a high autophagic capacity. Increased glucose consumption by tumor cells led to the activation of autophagy upon glucose shortage and contributed to the differentiation of normal fibroblasts into CAFs (Chaudhri et al., 2013). In contrast in another study, lysophosphatidic acid activated mTORC1 resulting in the attenuation of autophagy, which helped to sustain the glycolytic capacity of CAFs (Radhakrishnan et al., 2019). Pancreatic stellate cells differentiate into CAFs and were involved in sustaining alanine levels in pancreas cancer cells. Autophagy-dependent alanine secretion from CAFs fueled PDAC cells and resulted in the inhibition of glucose and glutamine usage. However, alanine was used in the production of lipids and non-essential amino acids, which enables independence of tumor cells from glucose and nutrient deficiencies (Sousa et al., 2016).

CAV1, a structural component of caveola consisting of sphingolipids, cholesterol, and a signal regulator, has been suggested to negatively regulate NADPH oxidase, an important ROS producer, in different ways (Chen et al., 2014). Loss of caveolin1 (CAV1) induces metabolic reprogramming in stromal cells via increased autophagy/mitophagy, mitochondrial dysfunction, and aerobic glycolysis. In addition, loss of CAV1 has been linked to the hyperactivation of the TGF-β signaling. To understand how TGF-β signaling pathway hyperactivation contributed to the reprogramming of CAV1 deficient CAFs, TGF-β ligands and TGF-β receptor I (TGF-β-RI) were overexpressed in fibroblasts. In this scenario, TGF-β promoted tumorigenesis by directing CAFs toward catabolic metabolism. In addition, TGF-β has compartment-specific effects on tumorigenesis. In fact, stroma-derived TGF-β could stimulate signaling in an autocrine manner, causing fibroblast activation and an increase in the expression of CAF markers such as α-SMA. In stromal cells undergoing metabolic reprogramming, a decrease in CAV1 expression and increased oxidative stress, autophagy, mitophagy were observed as well (Guido et al., 2012). On the other hand, activation of mitophagy was suggested to contribute to a decline in oxidative phosphorylation capacity. Under these conditions of aerobic glycolysis, ketone bodies and lactic acid were produced in abundance (P. Zhou et al., 2017).

Mechanistically, loss of CAV1 enhanced the expression of glycolytic enzymes, including PKM2 and LDH-B, which were associated with reverse Warburg (Bonuccelli et al., 2010). PKM1 expression increases the glycolytic activity of stromal cells, leading to an increase in lactate production and triggering tumor inflammation. PKM2 stimulates the production of the ketone body 3-hydroxybutyrate, which causes stromal cells to go into a pseudo-starvation mode and induce an NF-κB-dependent autophagic program. It was suggested that oxidative stress played a role in CAV1 depletion in TME. Loss of stromal fibroblast CAV1 by autophagic/lysosomal degradation created a “lethal tumor microenvironment” in a co-culture system, in response to oxidative stress induced by breast cancer cells (Martinez-Outschoorn et al., 2010; Hu et al., 2022). Another study reported that ROS produced by tumor cells reduced CAV1 expression in CAFs (Herrera et al., 2013). Upregulation of oxidative stress in CAFs was sufficient to cause genomic instability in neighboring cancer cells, possibly contributing to increased aggressive activity (Pavlides et al., 2010). In a parallel study conducted in oral squamous cell carcinoma, stromal cells expressing low Cav-1 levels were associated with high autophagic activity. Here too, L-lactate, ROS, and MCT4 secretion from autophagic CAFs led to anabolic growth of OSCC (Herrera et al., 2013).

Activation of an autophagic program could have pro-tumorigenic or anti-tumorigenic effects depending on the cell compartment (Lee et al., 2012; Zhu et al., 2014). For example, CAFs with activated peroxisome proliferator-activated receptor (PPAR) enhanced autophagy, glycolysis, and senescence compared to control fibroblasts. Enhanced stromal PPAR expression was associated with a 70% increase in l-lactate accumulation in the TME. The contentious function of PPAR has been shown to exhibit either an autophagy-induced pro-tumorigenic effect in CAFs or antineoplastic effects in epithelial cancer cells (Avena et al., 2013). In a bladder cancer TME model, the role of autophagy was investigated by co-culturing T24 cancer cells with CAFs. Proliferation, invasion, and aerobic glycolysis in T24 cells after co-culture with CAFs were examined. There was no discernible difference between the autophagy-inhibited and control groups, but enhanced autophagy in CAFs boosted the proliferation and invasion of T24 cells *in vitro*. The lactate concentration was higher in the autophagy-induced groups than in the controls. Their findings suggested that CAFs might control bladder cancer proliferation, invasion, and metabolic shift in an autophagy-dependent manner (Dong et al., 2021). Other studies supported the idea that CAFs’ activity deteriorated upon autophagy deficiency. For instance, diminished proline biosynthesis and collagen formation were associated with a lack of autophagy in CAFs. The study provided evidence that the production of mitochondrial NADP(H) by the enzyme NADK2 (NAD kinase 2, mitochondrial) was regulated by mitophagy, and cells with NADK2 deletion failed to synthesize proline and became proline auxotroph due to mitochondrial NADP(H) deficiency. Therefore, reducing mitophagy in the stroma resulted in decreased cancer cell growth and tumor weight *in vivo* in PDAC models (Bai et al., 2023).

Mass spectrometry-based profiling and pathway-based systems analysis was used to compare 21 primary CAFs with normal fibroblasts generated from adjacent non-neoplastic lung tissue. As a result of these analyses, they identified several pathways whose metabolite abundance may distinguish CAFs from normal fibroblasts globally. Notably, an increase in dipeptides has been observed in CAFs, which likely implies an increase in basal autophagic activity. Hence, increased autophagy may account for or contribute to the metabolic differences between CAFs and normal fibroblasts in lung cancer patients (Chaudhri et al., 2013).

Based on their roles in tumor promotion and inhibition, CAFs appear to have a dual nature and play a variety of important roles in the progression of cancer. In one study, authors depicted that depletion of stellate and parallel CAF population limits tumor growth in desmoplastic colorectal and pancreatic cancer metastasis but not in nondesmoplastic metastatic tumors. Tumor-promoting effects mediated by myofibroblastic CAF-secreted hyaluronan and inflammatory CAF-secreted HGF. However, myofibroblastic CAF-expressed type I collagen mechanically restrained the tumor spread (Bhattacharjee et al., 2021). In contrast to the previously described tumor-promoting effect of α-SMA+ CAFs, a study showed that they might also have a tumor-suppressing function. Deletion of Sonic Hedgehog (SHH) protein in a pancreatic cancer mouse model exhibited a reduced number of α-SMA+ myofibroblasts and following elevated angiogenesis, proliferation and undifferentiated thus aggressive phenotype (Rhim et al., 2014).

The development of cancer treatments will be assisted by understanding and focusing on the functional mechanism of CAFs, which includes the start of crucial signaling pathways including autophagy on tumor-associated phenotypes.

## The role of autophagy in CAFs and tumor-associated phenotypes

**FIGURE 3:**
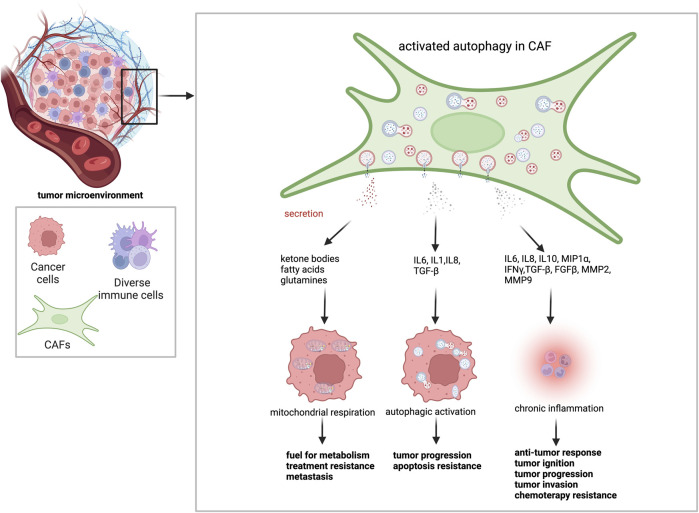
Autophagy activation in CAFs leads to changes in tumor behavior. Activated autophagy in CAFs provides a fertile environment for tumor cells. Through degradative autophagy, CAFs supply metabolic energy sources, such as ketone bodies, fatty acids, and amino acids, to cancer cells. It promotes mitochondrial respiration and leads to resistance to treatment and metastasis. Through secretory autophagy, CAFs secrete several cytokines and growth factors throughout the TME. While IL-6, IL-1, IL-8, and TGF-β contribute to cancer cell tumor metabolism, treatment resistance and metastasis by stimulating autophagy in cancer cells, IL-6, IL-8, IL-10, MIP1α, IFNγ, TGF-β, FGFβ, MMP2, and MMP9 contribute to the anti-tumor response, tumor ignition, tumor progression, tumor invasion, chemotherapy resistance.

Effects of CAFs on cancer progression heavily rely on their role in the regulation of extracellular matrix, as well as secreted growth factors, cytokines, and cell-to-cell interactions (Figure 3) (Table 2) (Wiseman and Werb, 2002; Kalluri, 2016).

**TABLE 2:** List of cancer-derived factors altering autophagy in CAFs.

Cancer type	Cancer-derived factor	Autophagy status in CAFs	Involved pathways	Effect on cancer cells	References
Breast cancer	CTF1	upregulation	STAT3 and AMPK signaling	promote invasion and metastasis	Akkoc et al. (2023)
Breast cancer	H_2_O_2_	upregulation	NF-κB and ROS signaling	fuel for mitochondrial biogenesis promote tumor growth	Martinez-Outschoorn et al. (2011)
Breast cancer	HIF1α	upregulation	NF-κB signaling	promote tumor growth	Chiavarina et al. (2010)
Breast cancer	TGF-β	upregulation	ROS signaling	promote invasion, migration and EMT	Huang et al. (2021)
Breast cancer	ROS	upregulation	miRNA upregulation	promote genomic instability	Pavlides et al. (2010)
Ovarian cancer	IL6	upregulation	STAT3 signaling	promote cell migration	Ferraresi et al. (2017)
Lung cancer	HMGB1	upregulation	NF-κB signaling	promote metastasis	Ren et al. (2021)
Oral squamous cell carcinoma	TGF-β	upregulation	SMAD	promote invasion and migration	Tan et al. (2021)
Nasopharyngeal	LMP1	upregulation	NF-κB signaling	promote proliferation, metastasis and radioresistancy	Wu et al. (2020)
Oral squamous cell carcinoma	Lnc-CAF	downregulation	IL33 upregulation	promote tumor growth	Ding et al. (2018)

CAFs remodel ECM and regulate tumor growth through the release of a series of ECM components (Orimo et al., 2005). Studies have demonstrated that ECM-degrading proteases, such as MMPs were produced by activated fibroblasts (Stetler-Stevenson et al., 1993; Sternlicht et al., 1999; Boire et al., 2005). MMPs help motility and promote invasive character in cancer cells. Autophagy deficiency in the stroma hampered the release of a set of cytokines to the TME, hence altering the tumor phenotype (Narita et al., 2011). For instance, genetic ablation of autophagy in stromal fibroblast significantly inhibited fundamental aspects of the stromal desmoplastic response, including collagen and proinflammatory cytokine secretion (IL6), extracellular matrix stiffening, and neoangiogenesis, in mouse mammary cancer models. Therefore, even though cancer cells were autophagy-competent, tumor development was attenuated, confirming the dependency of tumors on stromal autophagy (New et al., 2017).

CAV1 causes hyperactivation of TGF-β signaling and its target genes, such as CTGF, leading to the induction of autophagy and metabolic reprogramming of CAFs (Capparelli et al., 2012). To create a model illustrating the role of TGF-β signaling and autophagy, a study was conducted by overexpressing CTGF in MDA-MB-231 breast cancer cells and normal human fibroblasts. In the xenograft model used in this study, CTGF-overexpressing fibroblasts promoted the growth of co-injected MDA-MB-231 cells, without any increase in angiogenesis. CTFG-induced stromal autophagy was found to be responsible for the pro-tumorigenic effect of fibroblasts, yet a reversed scenario occurred when autophagic activity was elevated in cancer cells. Of note, deposition of ECM was elevated upon CTGF overexpression. Data suggested that the effect was not dependent on its role in the extracellular matrix, but rather on the capacity to trigger catabolic activity, which was autophagy (Capparelli et al., 2012). Migration stimulating factor (MSF) was a genetically truncated N-terminal isoform of fibronectin that was highly expressed in fetal fibroblasts and in cancer-associated fibroblasts. Recombinant overexpression of MSF in a fibroblast cell line was sufficient to induce myofibroblast differentiation via TGF-β signaling. In addition, MSF activated inflammation-associated transcription factor NF-κB, triggering autophagy and thereby promoting glycolytic metabolism (L-lactate production) in the TME. Consistent with the idea that glycolytic fibroblasts promote tumor growth via the high-energy fuel L-lactate, MSF-overexpressing fibroblasts significantly promoted tumor growth in breast cancer cells (Carito et al., 2012).

In our laboratory, we identified a factor that was secreted by cancer cells and influenced stromal fibroblasts. We discovered that CTF1/CT-1 (cardiotrophin-1) activated autophagy in fibroblasts and CAFs. Cancer-derived CTF1 triggered STAT3 phosphorylation and nuclear translocation, initiating transcriptional activation of essential autophagy genes. Furthermore, CTF1 activated both AMPK and ULK1 to further stimulate the autophagic activity in stromal cells. Moreover, CTF1-associated signaling was required for fibroblast activation as efficiently as the well-known CAF activator TGF-β. Strikingly, CTF1-dependent ACTA2/α-SMA accumulation, stress fiber formation, and fibroblast activation were dependent on stromal autophagy capacity. Furthermore, CTF1-induced stromal autophagy was necessary to facilitate migration and invasion of breast cancer cells. Analysis of patient-derived breast cancer specimens supported these *in vitro* observations. High expression of CTF1 in tumors correlated with abundant stroma, higher autophagic activity in CAFs. Moreover, higher levels of CTF1 expression in breast cancer tissues were associated with significantly higher rates of lymph node metastasis in patients. Proteomic data from CTF1-treated fibroblasts compared to controls linked CTF1 signaling with ECM remodeling and metabolic reprograming pointing out that similar to TGF-β, CTF1 plays a crucial role in fibroblast activation and CAF formation. In this context, autophagy as well, seems to play a key role (Akkoc et al., 2023).

In addition to being a catabolic mechanism, autophagy also contributes to the control of the ‘secretome’ and the surface proteome of cancer cells, which affect the outcome of the antitumor immune response. Secretory autophagy, as opposed to degradative autophagy, avoids lysosomal fusion and exports a variety of cytoplasmic substrates to the extracellular environment, including IL1, HMGB1. Tumor cell-released autophagosomes (TRAPs) were examples of secretory autophagy products. TRAPs affect the roles of neutrophils and B cells during immunological processes. The easy uptake of TRAPs by B cells can stimulate generation of IL10, which may inhibit T cell proliferation and antitumor responses (M. Zhou et al., 2016). ROS were produced as a result of the treatment of human neutrophils with TRAPs, which inhibited T-cell activation and proliferation (Gao et al., 2018). Hence, secretory autophagy may impair the antitumor immune cell response in the TME. Through secretory autophagy, cells in the TME can influence tumor behavior. Survival of cancer cells was aided by several cytokines, including IL6, IL8, IGF1, IGF2, and CXCL12 (Ngabire and Kim, 2017; Folkerts et al., 2019). Inflammatory and immunological responses were tightly linked to autophagy which was regulated by pro-inflammatory cytokines such as IFN, TNF-α, IL17, and cytokines from the IL1 family (Thuwajit et al., 2018; Song et al., 2019). Hence, CAFs secrete soluble factors through autophagy (Kimura et al., 2017). Exosomal transfer and paracrine signaling, by cytokines, including IL6 and GM-CSF, have been demonstrated as another mechanism of CAF-tumor cell interaction (von Ahrens et al., 2017). For example, secretory products of CAFs, such as IL6, directly influence control of autophagy and, as a result, the behavior of cholangiocarcinoma cells (Thongchot et al., 2021). In another study, fibroblasts co-cultured with breast cancer cells secreted IL13 and control the expression of BECN1 and LC3B through IKK/NF-κB (Li et al., 2017).

In addition to the above-mentioned outcomes on metabolism and immune system. Additionally, autophagy in CAFs also affects cancer stem cell pool in the TME. For example, breast cancer-initiating cells or stem cells were concentrated and rendered more tumorigenic in the stroma with highly active autophagy. It has been shown that autophagic CAFs secreted HMGB1, activated Toll-like receptor-4 (TLR4, the receptor of HMGB1 found in luminal breast cancer cells), and acted on the number of cancer stem cells.

## The role of autophagy in CAF-assisted treatment, drug resistance, recurrence, and tumor dormancy

A proteomic study demonstrated that the anti-cancer drug bortezomib could induce CAFs to generate high quantities of IL6, IL8, IGF-1, and TGF, which in turn activated oxidative stress and pro-survival autophagy in multiple myeloma (Frassanito et al., 2016). Usage of CAF-specific polyclonal antibodies (e.g., CAF-specific rabbit anti-CAF antibody, immunization of the rabbit with BFGF-activated fibroblast) resulted in the attenuation of tumor development in a mouse model of colon cancer (X. Li et al., 2018). Targeting CAFs and their secretion has been suggested as a potential alternative therapy in another tumor model. Conditioned medium from CAFs dramatically enhanced the IL6-mediated motility of cholangiocarcinoma (CCA) cells. Pre-treatment of CAF-conditioned medium with Resveratrol, a type of natural phenol produced from plants, stopped cancer cell motility and reversed EMT in migrating cells in the CCA model. Resveratrol suppressed the release of IL6 by CAFs. Moreover, they showed that this effect was linked to the induction of autophagy in cancer cells. Indeed, secretory products of CAFs may directly modulate autophagy and, subsequently behavior of CCA cells (Thongchot et al., 2018).

Targeting molecular mechanisms of autophagy in CAFs may counteract drug resistance in cancer and may provide another example of CAF-related cancer treatments. Interactions between CAFs and pancreatic cancer cells, and effects on chemotherapeutic drug responses were analyzed using drugs, such as α-cyano-4-hydroxycinnamate (CHC), metformin, and gemcitabine, as single or combined therapies. When autophagy was inhibited in CAFs, anti-proliferative effects of the chemotherapeutics increased *in vitro* tests and *in vivo* syngeneic cancer models (X. Zhang et al., 2018). However, cancer cells may also influence tumor-promoting properties of CAFs in an autophagy-dependent paracrine manner. For example, increased levels of stemness and autophagy have been identified in hepatic carcinoma cells cultured in CAF-conditioned media. Treatment of HCC cells with chloroquine (CQ) effectively reversed CAF-induced stemness, invasiveness, and metastatic potential. *In vivo*, Huh7 cells co-inoculated with CAF developed significantly larger tumors than controls. Moreover, blocking Huh7 cell autophagy with CQ significantly reduced the growth of xenografted tumor cells combined with CAFs (Zhao et al., 2019).

As mentioned earlier, several cellular cargos were exported via secretory autophagy, including cytosolic proteins and inflammatory mediators such as IL1, IL6, IL8, and IL18 (Ponpuak et al., 2015). Reducing autophagic activity in CAFs dramatically slowed the progression of neck squamous cell carcinoma through decreased secretion of IL6, IL8, and FGFB. SAR405, an inhibitor of the autophagy regulator Vps34, reduced xenograft development and increased the effects of conventional treatment (New et al., 2017). Similar outcomes have been demonstrated for different cancers, including breast, ovarian, liver, colorectal, and pancreatic cancer, *in vitro* co-cultured models and *in vivo* mice xenografted models as well as inpatient-derived tissue analyses (Wang M. et al., 2017; Ngabire and Kim, 2017). Combined use of the drug CHC with metformin, led to the suppression of autophagy in CAFs and inhibited cancer cell growth, *in vitro* and in syngeneic pancreatic cancer models (Shan et al., 2014). CAFs were shown to generate IGF1/2, CXCL12, and α-hydroxybutyrate and increase the amount of ROS after radiation in lung cancer and melanoma cell xenograft models *in vitro* and *in vivo*. An increased protein phosphatase 2A (PP2A) activity in this context, resulted in the inhibition of mTOR activation and boosted autophagy in cancer cells after radiation. Hence, lung cancer relapse following irradiation was associated with CAF-derived IGF1/2, CXCL12, and-hydroxybutyrate and autophagic activity was instrumental in the elimination of radiation-induced DNA damage in lung cancer. Inflammatory CAFs were attenuated by the inhibition of IL1a signaling and facilitating chemotherapy resistance in rectal cancer (Nicolas et al., 2022). In another example, bone marrow stroma-derived exosomes loaded with miR 222/224, miR-127, and miR-197 were found to induce dormancy in breast cancer by suppressing CXCL12 (Lim et al., 2011).

## Conclusion

Fibroblast activation was fundamental in wound healing, inflammation, and extracellular matrix remodeling. Moreover, activated fibroblasts play an undeniable role in carcinogenesis. The idea was first proposed by Rudolph Virchow in the late 1850s as the ‘theory of irritation’. According to Virchow, irritation and subsequent chronic inflammation were among the main factors in neoplastic transformation (Marzella et al., 1981). Almost a century later, tumors were called ‘non-healing wounds’, highlighting similarities between cancers and wound healing mechanisms (Dvorak, 1986). In fact, as presented above, fibroblasts that were activated in a malignant context, CAFs in the TME, provoke and support cancer formation and progression, including migration, metabolic switch, angiogenic cytokine signaling, and the plasticity of cancer stem cells, and furthermore, drug resistance and resistance to tumor immunity mechanisms.

Studies attributed variable roles to autophagy in the TME. A grand majority of the studies provided evidence about alterations of autophagy in the TME in a stress-responsive manner. Cancer cells typically use autophagy to survive upon demanding tumor-related circumstances, such as hypoxia, nutritional restriction, or oxidative damage. Under these conditions, autophagy helps tumor cells to adapt and endure environmental stress. Similar stress resistance-related advantages of autophagy were reported at different stages of metastasis, including migration and invasion, extravasation, circulation, and colonization. Hence overall, recycling of cytoplasmic materials and damaged organelles via autophagy enhances metastatic recurrence and provides cancer cells with building blocks and energy sources that were required for survival (Kocaturk et al., 2019).

As we summarized above, autophagy plays a role in remodeling the TME and regulates tumor-stroma interactions. Studies have identified several factors e.g., ROS, CTF1, TGF-β that were derived from cancer cells and leading the activation of autophagy in the stroma. However, not all were carefully studied autophagic mechanisms besides documenting the effect on its activity. Autophagic activity has been strictly controlled by numerous transcriptional and post-translational events. In addition, it was also equally important to state the changes in autophagy such as alteration of the autophagosome biogenesis and/or degradative autophagic capacity. Hence, careful dissection of autophagy in CAF formation and its effect on cancer progression leads to the identification of smart agents to differentially target autophagy and eventually maintain better clinical outcomes.

Most of the studies have been conducted on tumors that were rich in stroma components. According to those studies, the importance of the involvement of CAFs in cancer phenotype seems accepted. However, most of the studies lack of multi-variant studies including the investigation of multiple stromal components e.g., immune cells, CAFs, cancer, etc. and have focused on single cell types and/or molecules. Still, there is no consensus on the modulatory factors or deregulated pathways that have been postulated as important for CAF and cancer crosstalk. Autophagy, as it plays a central role in almost all the above-mentioned components and cancer progression, seems to have great potential among all other pathways.

Therefore, characterizing the molecular mechanisms of fibroblast activation, CAF formation, and the involvement of autophagy in TME is important for the identification of new molecules and pathways for potential therapeutic targets that can make a significant impact on patients.
